# Optimizing the diagnostic work-up of acute uncomplicated urinary tract infections

**DOI:** 10.1186/1471-2296-9-64

**Published:** 2008-12-08

**Authors:** Bart J Knottnerus, Patrick JE Bindels, Suzanne E Geerlings, Eric P Moll van Charante, Gerben ter Riet

**Affiliations:** 1 Department of General Practice, Academic Medical Center – University of Amsterdam, Amsterdam, the Netherlands; 2 Department of Infectious Diseases, Tropical Medicine & AIDS, Center for Infection and Immunity Amsterdam (CINIMA), Academic Medical Center – University of Amsterdam, Amsterdam, the Netherlands; 3 Horten Centre, University of Zurich, Zurich, Switzerland

## Abstract

**Background:**

Most diagnostic tests for acute uncomplicated urinary tract infections (UTIs) have been previously studied in so-called single-test evaluations. In practice, however, clinicians use more than one test in the diagnostic work-up. Since test results carry overlapping information, results from single-test studies may be confounded. The primary objective of the Amsterdam Cystitis/Urinary Tract Infection Study (ACUTIS) is to determine the (additional) diagnostic value of relevant tests from patient history and laboratory investigations, taking into account their mutual dependencies. Consequently, after suitable validation, an easy to use, multivariable diagnostic rule (clinical index) will be derived.

**Methods:**

Women who contact their GP with painful and/or frequent micturition undergo a series of possibly relevant tests, consisting of patient history questions and laboratory investigations. Using urine culture as the reference standard, two multivariable models (diagnostic indices) will be generated: a model which assumes that patients attend the GP surgery and a model based on telephone contact only. Models will be made more robust using the bootstrap. Discrimination will be visualized in high resolution histograms of the posterior UTI probabilities and summarized as 5^th^, 10^th^, 25^th ^50^th^, 75^th^, 90^th^, and 95^th ^centiles of these, Brier score and the area under the receiver operating characteristics curve (ROC) with 95% confidence intervals. Using the regression coefficients of the independent diagnostic indicators, a diagnostic rule will be derived, consisting of an efficient set of tests and their diagnostic values.

The course of the presenting complaints is studied using 7-day patient diaries. To learn more about the natural history of UTIs, patients will be offered the opportunity to postpone the use of antibiotics.

**Discussion:**

We expect that our diagnostic rule will allow efficient diagnosis of UTIs, necessitating the collection of diagnostic indicators with proven added value. GPs may use the rule (preferably after suitable validation) to estimate UTI probabilities for women with different combinations of test results. Finally, in a subcohort, an attempt is made to identify which indicators (including antibiotic treatment) are useful to prognosticate recovery from painful and/or frequent micturition.

## Background

### General background

An acute uncomplicated urinary tract infection (UTI, cystitis) is defined as an infection of the lower urinary tract (bladder) in an otherwise healthy, nonpregnant, adult woman without known anatomical or functional abnormalities of the urinary tract. [1-4] A U.S. study reported a 60% lifetime risk of physician-diagnosed UTI.[5]

The main symptoms suggesting a UTI are dysuria and urinary frequency, whereas vaginal irritation and discharge have been reported to reduce the probability of a UTI being present.[6] Besides a UTI, an important cause of dysuria and frequency is a *Chlamydia *urethritis. Other infectious diseases in the differential diagnosis of a UTI are other types of urethritis (e.g. gonorrhoea), vaginitis, interstitial cystitis and genital herpes. [6-8] Since a UTI differs from these diseases in its natural course and treatment, a correct diagnosis is essential.

Empirical treatment of symptomatic women has been reported to be cost-effective.[9,10] However, only half of symptomatic women are found to have a UTI if defined by ≥ 10^5 ^colony-forming units (CFU)/ml.[11,12] Moreover, bacterial resistance to commonly used antibiotics (e.g. trimethoprim and fluoroquinolones) is rising, suggesting the need for more evidence-based prescribing. [13-16] Better diagnosis of UTIs might prevent women from being unnecessarily treated with antibiotics and be even more cost-effective.

### Diagnostic work-up

Diagnosis of UTIs in general practice consists of various medical history questions and laboratory investigations, of which nitrite and leukocyte esterase (LE) dipstick tests, microscopic examination of urinary sediment, and dipslide are the ones most widely used.

Most diagnostic tests for UTIs have been studied before in single-test evaluations, implying that a test is compared to the urine culture, not taking into account the distribution of other test results in the population studied. [17-19] In practice, however, the diagnostic work-up is inherently multivariable, and test results are mutually dependent. [20-36] Therefore it cannot be generally expected that a test's sensitivity is invariant over different degrees of prior testing. For example, the sensitivity of the nitrite dipstick test will be affected by the information contained in the diagnostic profile resulting from prior test results.[37]

The primary objective of the Amsterdam Cystitis/Urinary Tract Infection Study (ACUTIS) is to determine the (additional) diagnostic value of relevant tests from patient history and laboratory investigations, taking into account their mutual dependencies. Next, an easy to use, multivariable diagnostic rule (clinical index) will be derived. After suitable (external) validation, GPs may use the (validated) rule to estimate UTI probabilities for women with different combinations of test results.

Little et al. and McIsaac et al. described similar work in a UK and a Canadian population, respectively.[38,39] However, different inclusion criteria were used (see further) and urine samples were transported as in routine practice before cultures were made, whereas in ACUTIS, to assure reliability of the cultures (= reference standard), urine samples are being refrigerated until being cultured. Furthermore, both earlier studies did not assess diagnostic values of the sediment and dipslide investigations.

Other similar studies.[40,41] were performed before the European Urinalysis Guidelines.[42] recommended a cut-off value of 10^3 ^cfu/ml for significant *Escherichia coli *bacteriuria and therefore only used the traditional cut-off value of 10^5 ^cfu/ml. Besides, they did not assess dipstick investigations and/or were not performed in a primary care setting.[41]

### Prognosis and natural history

Placebo arms of randomized controlled trials suggest that 25 to 50% of women presenting with a combination of UTI-symptoms and bacteriuria will have recovered in one week without using antibiotics. [43-45] Using data from ACUTIS, we will try to make a prognostic index. Using the prognostic index, a GP can estimate the 7-day course for women with different combinations of test results and decide which women are most likely to benefit from antibiotic treatment.

## Methods

### Setting

ACUTIS is performed in general practices and primary health care centers in Amsterdam and surroundings, which altogether comprise 20 practices of average size (total source population = 46,000 patients).

### Design

In a prospective cohort, each participating patient will be followed for 7 days. At baseline, potentially relevant signs and symptoms will be recorded and potentially relevant laboratory tests (dipstick, sediment and dipslide) will be performed, along with a urine culture as reference test. Details are described below.

### Study population

Eligible are female patients over 12 years of age, contacting their GP with painful and/or frequent micturition. The symptoms may have been present for a maximum of 7 days. Although not being adult and therefore not strictly meeting the definition of acute uncomplicated urinary tract infection, patients between the age of 12 and 18 are eligible, as their urogenital tract is expected to be anatomically similar to that of adult women.

Exclusion criteria are: pregnancy, lactation, signs of pyelonephritis, being immunocompromised (e.g. receiving radiotherapy or immunosuppressants, being HIV infected) with the exception of diabetes mellitus, having used antibiotics for another indication than UTI or having undergone a urological procedure in the past two weeks, and known anatomical or functional abnormalities of the urogenital tract.

To ensure a clinical domain in which the diagnostic index may be expected to perform as reported and to enhance generalizability in time and place, we use clearly formulated eligibility criteria. This differs from similar studies, in which patients were eligible when GPs "suspected" a UTI, based on their personal judgement.[38,39]

### Informed consent

A letter with information about the study is provided to all patients. Participating women must provide written informed consent prior to enrollment. For patients under the age of 18 written parental authorization is required.

### Sample size

When the events per variable (EPV) rule.[46,47] is applied on a number of 15 possible diagnostic indicators and taken into account that some indicators will have to be modeled as dummy variables, about 200 women with a UTI are needed for this study (although a more recent simulation study suggests that the traditional EPV figure may be an overestimate.[48], so that less patients may be needed for our study). Assuming that the probability of a UTI (if defined by ≥ 10^5 ^CFU/ml) in eligible women is approximately 50%.[11,12] (range 40–60%), between 333 and 500 women will have to be included in the study. According to a UTI incidence in The Netherlands of 68 per 1000 women per year.[49], 157 women per year are diagnosed with a UTI in a general practice of average size. Assuming that 80% of all eligible women will not take part in the study, 16–17 practices of average size that include patients for a period of 1 year are needed (500/(157 × (1-0.8)) = 16.03).

### Clinical assessments (see table 1)

**Table 1:** Assessment schedule

	**GP or GP assistant**	**Patient**
**day 1 (baseline)**	Check availability	
	Obtain informed consent	
		Collect urine at GP surgery
	Perform dipstick and dipslide (and optionally sediment)	Fill in questionnaire
	Send urine to laboratory (refrigerated) for culture and sediment	

**day 2**	Examine dipslide	

**day 1 till day 7**		Record symptoms in diary

**day 8**	If patient did not use antibiotic: collect urine and send for culture	

After eligibility has been confirmed and informed consent has been obtained, urine is collected in a sterile container. Instructions for the collection method may be given at the GP's discretion, but are not necessary, as they have been reported to have no consequences for the extent of contamination. [50-52] We insist that the urine sample is collected at the GP surgery, since collection at home will cause at least some specimens to remain non-refrigerated for too long, thus rendering urinalysis unreliable.[53]

After urine collection, patients are asked to fill in a detailed questionnaire to record presence and severity of signs and symptoms (see additional file 1). Furthermore, they are given a special diary, in which they are to record clinical symptoms and any use of antibiotics for 7 days.

The GP or GP assistant performs a dipstick test (Multistix^® ^5, Siemens Medical Solutions Diagnostics™) and a dipslide test (Uricult^® ^classic, Orion diagnostica™). Dipsticks will be assessed automatically by a Clinitek Status^® ^analyzer (Siemens Medical Solutions Diagnostics™) to reduce inter-observer variation.

Immediately after dipstick and dipslide investigations have been performed, urine samples are stored in a refrigerator at the GP surgery. Within 8 hours they are transported to two laboratories (see below) at 4°C by a specialized courier service.

GPs are free to perform a urinary sediment investigation, the results of which will then be documented. However, in the statistical analysis we will assess the diagnostic value of the urinary sediment investigation performed by experts at the Laboratory for Clinical Chemistry of the Academic Medical Center in Amsterdam. Only if the sediment investigation proves to be useful under the optimal conditions of the laboratory, we will repeat it under daily practice conditions in a sub-study to be carried out after the main study has been completed (see further).

The Laboratory for Medical Microbiology of the Academic Medical Center in Amsterdam performs a urine culture, which serves as the reference standard. Trained laboratory technicians, who have no information on previous test results from history taking and/or urinalysis, will make a culture according to the standard loop method.[53], interpret the culture results and determine resistance patterns.

Patients who have not used antibiotics since their study entry will be asked to provide another urine sample for culturing at day 7 (see below).

Urine samples with a culture result ≥ 10^2 ^CFU/ml will be stored refrigerated for possible later use (e.g. identification of bacterial strains).

### Data entry

The information obtained from patient history and laboratory investigations will be collected and entered into a structured database by a qualified commercial data-entry service, which guarantees a 97% accuracy at character level. For crucial variables (e.g. culture results) there will be double (independent) entry (99.7% accuracy at character level).

## Statistical analysis

### Data screening

The data will be screened for data entry errors, extreme values, and missing values using simple tables, plots, and dedicated commands to identify (multivariable) outliers. Errors will be corrected. Instead of deleting extreme cases, we shall consider to suitably truncate extreme values where their influence on models appears too strong.[54]

### Missing values

Multiple imputation using chained equations will be used to impute missing values.[55,56]

### Dependent variable

The results of the urine culture will be used as the dependent variable. A cut-off value of 10^3 ^CFU/ml will be used to define a positive culture, as is recommended by the European Urinalysis Guidelines.[42] We will explore how sensitive the model is to the use of different cut-off values for defining a positive culture.[6,12,57-60]

Theoretically, false positive reference standard results may occur if women with asymptomatic bacteriuria are included in the study when they visit their GP with complaints similar to UTI symptoms but caused by a different disease (e.g. vaginitis). In the community, the prevalence of asymptomatic bacteriuria has been reported to be 1 to 5% in premenopausal, nonpregnant women, growing with increasing age to >10% in women over 70 years of age.[61]

### Variable selection strategy

#### General objectives

We aim to generate two models (diagnostic indices). First, a model which assumes that patients attend the GP surgery. Second, a model based on telephone contact only, since in daily routine this is very often the first and only contact with the patient.

Finally, using different threshold probabilities for 'no further action' (e.g. advice to increase the intake of water, 'wait and see') and prescription of an antibiotic, respectively, we will develop a diagnostic test algorithm such that patients may undergo the least number of possible tests. That is, if, regardless of its results, a certain test can be shown not to take a patient across one of the abovementioned thresholds for specific clinical action, that test may be informative, but not sufficiently so to change management. Therefore it is superfluous. It is possible that a 'telephone only' model provides sufficient certainty for patients with specific combinations of patient history items, while for other patients urinalysis at the GP surgery is needed to decide on management correctly.

#### Variable selection

At the time of writing, a failsafe model selection strategy on which all statisticians and other experts in diagnostic and predictive modeling agree does not appear to exist. However, in his book 'Regression modeling strategies (2001)'[62], Harrell made some general proposals for researchers to tailor to the specific circumstances. We intend to use penalized logistic regression, and Tibshirani's *lasso *(least absolute shrinkage and selection operator)[63] in particular to combine the requirements of counteracting over-optimism (shrinkage of regression coefficients) while leaving the opportunity that some coefficients are set to zero, which serves the requirement of a parsimonious model. Bootstrapping will be used to estimate the penalization coefficient.[62] As a form of sensitivity analysis, we shall also explore bootstrapped stepwise regression[64,65] to see how well these approaches concur. We will avoid univariable preselection of predictors. The linearity assumption will be checked for all continuous predictors.

Discrimination of the model(s) will be visualized in high resolution histograms and summarized as 5^th^, 10^th^, 25^th ^50^th^, 75^th^, 90^th^, and 95^th ^centiles of these, Brier score and the area under the receiver operating characteristics curve (ROC) with 95% confidence intervals (overall discrimination).[66]

Using the regression coefficients of the independent diagnostic indicators, an easy to use, multivariable diagnostic rule (clinical index) will be derived, consisting of relevant tests and their diagnostic values.

## Substudies

### Prognosis

Using our diagnostic rule, the probability of a positive culture at baseline can be estimated. For a clinician it may also be relevant to learn more about a patient's prognosis and how this is affected by antibiotic medication. In previous studies 25 to 50% of women presenting with UTI symptoms and/or bacteriuria have been reported to recover spontaneously within one week. [43-45] It would be useful to know whether these patients, for whom antibiotics are not indicated, can be identified at baseline based on their clinical profiles.

In ACUTIS, all patients are asked to record their symptoms and possible antibiotic use in a special diary for 7 days. To learn more about the natural course of UTI, we will offer eligible women the opportunity to postpone the use of antibiotics. For patients who have not used antibiotics after 7 days, we shall try to secure a second urine specimen for culture. As a result, two prognostic models may be developed, with clinical and bacteriological cure after one week serving as dependent variables, respectively. Regression analysis will be used to assess the effects of antibiotic treatment using all cogent potential confounders to counteract possible confounding by indication as much as possible. If in specific subdomains of women no effect of antibiotics is found, randomised trials may be indicated to confirm such findings. If, on the other hand, an effect is found, the effect may be non-existent in particular subgroups. However, the power to detect such subgroup effects (or a lack of it) is probably somewhat limited. Women with milder symptoms may be more likely to defer antibiotics. We consider this not to be a great problem, since this is the subdomain of patients in which doubt about the value of antibiotics is greatest. Placebo-controlled randomized clinical trials in the subdomain of patients with severe dysuria and/or frequency are likely to incur ethical problems. If our results are promising, however, an RCT may be performed in the future (possibly by using a delayed prescription approach. [67-69]).

### Sediment

As mentioned before, urinary sediment investigation will be performed under protocollized circumstances at the Laboratory for Clinical Chemistry. It could be argued that this should be done in the GP surgery, as this is more in accordance with common practice. However, if urinary sediment proves to be useful under the optimal conditions of the laboratory, it will be repeated under daily practice conditions in a sub-study after the main study has been completed. The originally obtained sediment data can then be replaced in our model by the daily practice results, using Deming (orthogonal) regression analysis. [70-76] Briefly, this is a method that can be used to estimate the relationship between two measurements with proportional errors, in this case sediment under optimal conditions and sediment under daily practice conditions. The data collection of this sediment sub-study will take place during a specially organized training session, where sediment scoring results of GPs and/or GP assistants will be measured before and after training by experts. Sediment investigation on the same samples will be performed by experts under optimal conditions (= reference standard). The before-training measurements as well as the after-training measurements will be compared with the expert measurements using Deming regression.

### Chlamydia/gonorrhoea

Apart from a UTI, dysuria and urinary frequency in sexually active women may be a result of a urethritis caused by *Chlamydia trachomatis *or *Neisseria gonorrhoeae*. Therefore a polymerase chain reaction (PCR) for *Chlamydia trachomatis *and *Neisseria gonorrhoeae *will be done in each urine sample. An attempt will be made to extend our diagnostic rule for *Chlamydia trachomatis *and *Neisseria gonorrhoeae *infections in women with painful and/or frequent micturition. To attain this, we will perform a polytomous logistic regression analysis, a technique that is used to simultaneously estimate probabilities of multiple diagnoses. [77-79] As a result, a diagnostic model with three outcomes (UTI, *Chlamydia trachomatis*, and *Neisseria gonorrhoeae*) can be derived.

## Discussion

Whereas most previous studies on diagnosis of UTIs are single-test evaluations, we will perform a multivariable analysis and develop an easy to use diagnostic rule for general practice. This will allow efficient diagnosis of UTIs, using only those diagnostic indicators which have proven added value to rule in or rule out UTI. For a large number of women with painful and/or frequent micturition, additional urine investigations might prove to add no useful information to what is already known from specific patient history questions.

This implies that in these cases the diagnostic procedure can be completed by telephone, which makes life easier for GP and patient alike. Another possibility is that patients, before contacting their GP, use a simplified questionnaire (based on the study results), available e.g. from the Internet or from their GP's office. As a result, the diagnostic work-up will be more cost-effective and less time-consuming. Besides, since use of dipslide and culture might be reduced, there may be less time delay in UTI diagnosis.

In sub-studies we will try to extend our diagnostic rule to diagnosis of *Chlamydia trachomatis *or *Neisseria gonorrhoeae*. Furthermore, prognostic indicators (potentially including antibiotics) of the 7-day course of painful and/or frequent micturition will be assessed in our cohort study.

We expect to be able to submit the first results of ACUTIS no later than by the end of 2009.

## Competing interests

The authors declare that they have no competing interests.

## Authors' contributions

BK participated in the design of the study, drafted the manuscript and will coordinate data collection, statistical analysis and reporting of the results. PB, SG, EM and GtR participated in the design, helped to draft the manuscript and will support or participate in data collection, statistical analysis and reporting of the results. GtR conceived the basic design and the main objectives of the study.

## Pre-publication history

The pre-publication history for this paper can be accessed here:



## Supplementary Material

Additional File 1 **Questionnaire.** At presentation at the GP surgery, patients are asked to fill in this questionnaire to record presence and severity of signs and symptoms. Click here for file
