# Supplementary material for: Optimizing the diagnostic work-up of acute uncomplicated urinary tract infections
Source: BMC Fam Pract. 2008 Dec 8;9:64. doi: 10.1186/1471-2296-9-64 (PMC2607275; doi:10.1186/1471-2296-9-64)
Supplement: Additional File 1 — Questionnaire. At presentation at the GP surgery, patients are asked to fill in this questionnaire to record presence and severity of signs and symptoms. [file 1471-2296-9-64-S1.doc]

**STRICTLY CONFIDENTIAL
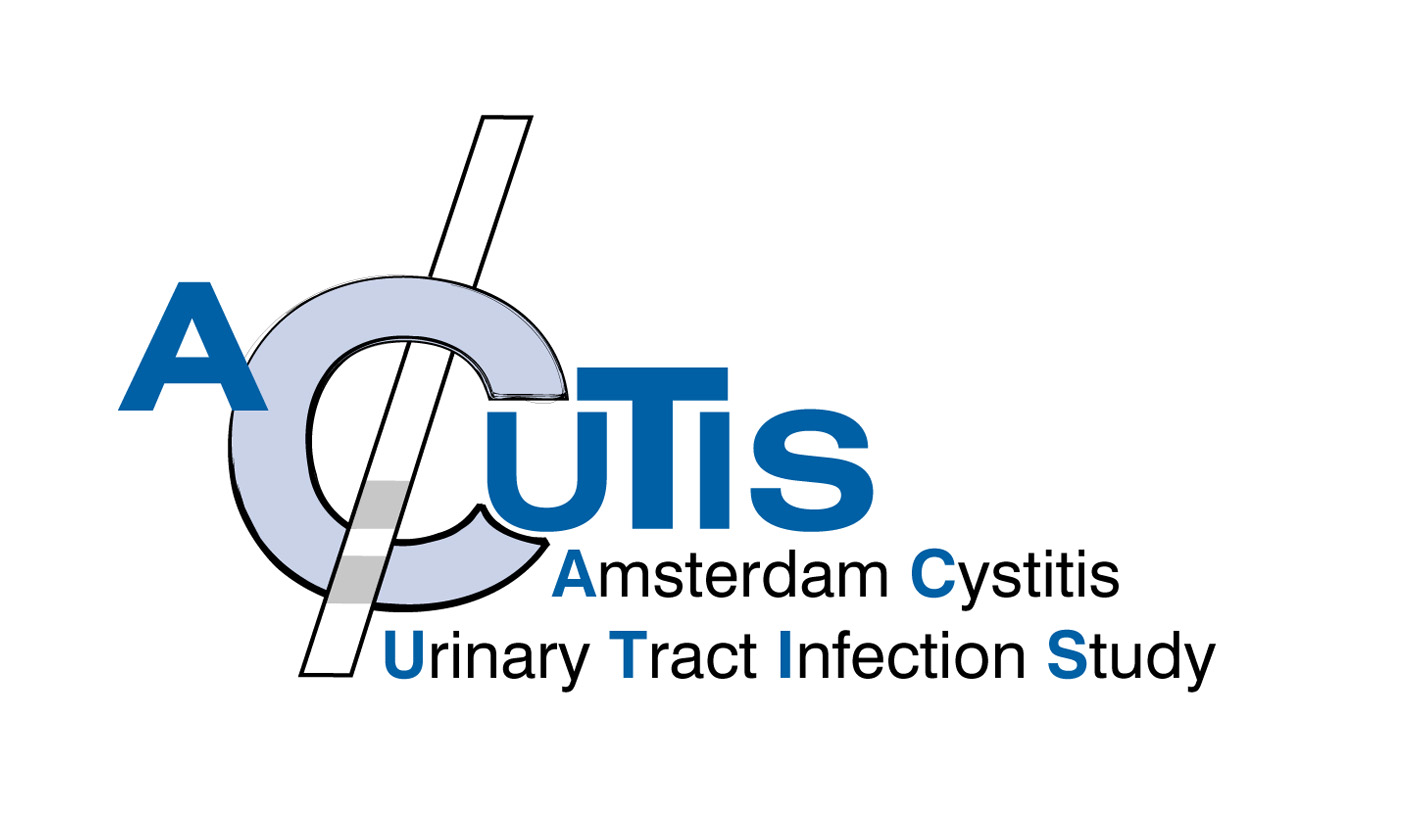
**

**DiagnosIS OF UNCOMPLICATED urinARY TRACT INFECTIONS IN general PRACTICE**

**PATIENT FORM**

## To be completed by the patient

**Date of visit: |___|___| - |___|___| - |___|___|___|___|**

**d d m m y y y y**

**Patient code: |___|___|___|___|**

**Name of practice/health centre:**

Academic Medical Center - University of Amsterdam

Division of Clinical Methods and Public Health

Department of General Practice

Investigators:

B.J. Knottnerus, General Practice

G. ter Riet, General Practice

S.E. Geerlings, Internal Medicine / [Infectious Diseases, Tropical Medicine and AIDS](http://www.amc.nl/index.cfm?pid=800)

E.P. Moll van Charante, General Practice

prof. P.J.E. Bindels, General Practice

**CONTENTS** *Page*

### DECLARATION OF CONSENT *3*

**GENERAL INFORMATION AND INSTRUCTIONS** *4*

**QUESTIONNAIRE** *5*

**DECLARATION OF CONSENT**

I hereby declare that I have been sufficiently informed about the ACUTIS research project on diagnosis of bladder infections, which is performed by the Department of General Practice of the Academic Medical Center, Amsterdam.

Yes, I agree to participate in the research project on diagnosis for urinary tract infections.

No, I will not participate in the research project on diagnosis of urinary tract infections.

*Please cross one of the squares.*

Date: **|___|___| - |___|___| - |___|___|___|___|**

**d d m m y y y y**

Signature Signature of parent or legal guardian (if required)

**GENERAL INFORMATION AND INSTRUCTIONS**

- Fill in the questionnaire with a **blue** or **black** pen.
- Make sure that your answers are legible, preferably in block letters.
- Give only one answer to each question, unless the questionnaire states otherwise.
- Answer **all** the questions
- Place a cross or a tick in the box next to your chosen answer.
- Enter any dates in dd-mm-yyyy format (e.g. 01-01-2006).
- Do not use abbreviations.
- If you place a cross or a tick in the wrong box, you can rectify this by placing a cross or a tick in the correct box and **encircling** it.
- Only explain your answer when specifically asked to do so.

**QUESTIONNAIRE**

You have agreed to participate in a research project on bladder infections. We would like you to answer the questions in this form. It will take approximately 5 minutes. Your answers will be processed anonymously.

Please read each question carefully. Place a cross or a tick in the box that best describes your situation. There are no right or wrong answers. Please do not skip any questions and fill in only one answer to each question, unless otherwise requested. If you need help you can turn to your GP or his/her assistant.

**1. Personal data**

Name and initials: ………………………………………………………

Telephone no.: ………………………………………………………

*N.B. The personal data will be used only if we need to contact you about questions that have not been (clearly) answered. Your name and phone number will be deleted as soon as they are no longer needed.*

# 2. Symptoms related to bladder infection

**2.1** For how many days have you been experiencing your present urinary symptoms?

less than one day

one day

longer, namely ……………….. days (state the number of days)

**2.2** Is it painful when you urinate?

no a little painful  quite painful  very painful

**2.3** Do you feel a burning sensation when you urinate?

no a little  quite much  very much

**2.4** Do you have to urinate more often than usual?

no a little more often  much more often  very much more often

**2.5** Do you urinate smaller amounts than usual?

no a little smaller  much smaller  very much smaller

**2.6** Do you have the feeling that you can’t urinate properly (you can’t completely empty your bladder)?

no a little  quite much  very much

- 1. Do you feel feverish?

no a little feverish  quite feverish  very feverish

**2.8** Are there moments when the urge to urinate is so strong that you are (almost) unable to control it?

no sometimes  quite often  very often

**2.9** Is there blood in your urine?

no sometimes  quite often  very often

**2.10 a)** Do you suffer (more than usual) accidental urine loss?

no, go to Question **2.11** sometimes  quite often  very often

**b)** When do you suffer accidental urine loss? (You may give more than one answer.)

when I laugh or cough, or during physical activity

when I feel the need to urinate (can’t get to the toilet on time)

other, namely ……………………………………………………..

**2.11** Do you feel an urge to urinate when you don’t really have to (false urge)?

no sometimes  quite often  very often

**2.12 a)** Do you have (more than usual) pain in your lower abdomen?

no, go to Question **2.14** a little  quite much  very much

**b)** When do you feel this pain?

all the time  only during urination  other, namely …………...

**2.13 a)** Do you have (more than usual) pain in your back?

no, go to Question **2.14** a little  quite much  very much

**b)** When do you feel this pain?

all the time  only when I urinate  other, namely …………...

**2.14** Does your urine have a bad smell?

no a little  quite bad  very bad

**2.15** Do you feel itching or irritation in or around the vagina?

no a little  quite much  very much

**2.16** Are you experiencing (heavier) vaginal discharge?

no a little  quite much  very much

**2.17** How often has a doctor diagnosed you with a bladder infection?

never  once  2-5 times  more than 5 times

**2.18** **a)** How often in your life have you experienced similar symptoms without consulting a doctor?

never  once  2-5 times  more than 5 times

**b)** What remedies (if any) did you yourself take to treat the symptoms?

cranberries (juice or tablets)  vitamin C  pain killers

other, namely …………………………………………………………

not applicable

**2.19** How often have you had a bladder infection **in the past year**?

never

once

twice

more than twice

don’t know

**2.20** Do you think that you have a bladder infection at this moment?

yes  no  don’t know

**3. Sexuality**

It is probable that sexual factors play a role in urinary tract infections. It is therefore important that you answer the following questions.

**3.1**  Are you sexually active?

yes  no, go to Question **4.1**

**3.2 a)** How often did you have sex in the week before the start of your present urinary symptoms?

……. times

**b)** Is that less often, the same, or more often than usual?

less often  the same  more often  not applicable

**3.3** Do you normally urinate directly after having sex?

never  sometimes  usually  always

**3.4** Do you have pain during sexual activity?

no a little  quite much  very much  not applicable

**4. General questions**

**4.1** What is your date of birth?: **|___|___| - |___|___| - |___|___|___|___|**

**d d m m y y y y**

**4.2** What is your marital status?

married  permanent partner, co-habiting

permanent partner, not co-habiting  single

other, namely ………………………………………………..................

**4.3** What is your economic status? (You may give more than one answer.)

schoolgoing or student  employed (full- or part-time)

housewife  unemployed

(partly) unfit for work  (early) retirement

**4.4** What population group do you consider yourself to belong to?

Dutch

Turkish

Moroccan

Surinam

Antillean or Aruban

other, namely …………………………………

**4.5** How many full-term pregnancies have you had?

………..

**4.6** How many times have you given birth vaginally?

………..

**4.7** How tall are you in centimetres?: |___|___|___| **cm**

**4.8** What is your weight in kilograms?: |___|___|___| **kg**

**4.9** How would you describe your health **in general**?

very good  good  reasonable  moderate  poor

**4.10** Do you have diabetes?

yes  no

**4.11** Are you taking pain killers at the moment to ease your urinary symptoms?

yes  no

**4.12** Are you taking cranberries at the moment (juice or tablets)?

yes  no

**4.13** Are you taking Vitamin C at the moment?

yes  no

**4.14 a)** Did or does any member of your immediate family (mother, sister, daughter) suffer a bladder infection more than twice a year on average?

yes  no, go to Question **4.15**

**b)** Which member of your immediate family suffered or suffers a bladder infection more than twice a year on average? (You may give more than one answer.)

mother  sister  daughter

**4.15** Are you menstruating at the moment?

yes  no

**4.16** Was your last menstruation longer than a year ago?

yes  no

**4.17** You have just handed in a urine sample for testing. How much time passed between the sample you produced for testing and the previous urination?

less than 4 hours  4 hours or more

- 1. How much do your urinary symptoms bother you in:
- your work or other daily activities?

not at all  hardly  moderately  much  very much

- social activities (e.g. visiting family and friends)?

not at all  hardly  moderately  much  very much

**4.19** How many days of work/studying/school have you missed **due to your current urinary symptoms**?

……… days  not applicable

**4.20** A bladder infection is confirmed in only half of the women who have the same symptoms as you at this moment. The final diagnosis can only be made by making a urine culture. However, the results of the culture are not known until **one day or a few days** later. Antibiotics may only be useful if you do actually have a bladder infection.

**a)** Would you be willing to delay taking antibiotics until you know for sure that you have a bladder infection?

yes  no  don’t know

**b)** If so, how many days would you be willing to wait?

…….. days

**You have reached the end of the questionnaire. Please check if you have answered all the questions.**

**Thank you for your participation.**
